# New Appliances and Things Medical

**Published:** 1897-01-23

**Authors:** 


### NEW APPLIANCES AND THINGS MEDICAL.

[We shall be glad to receive, at our Office, 28 & 29, Southampton Street, Strand, London, W.C., from the manufacturers, specimens of all new preparations and appliances which may be brought out from time to time.]

### HOLYROOD TABLE WATER.

(J. F. MACFARLAN AND CO., EDINBURGH.)

The composition of this new table water is founded on the analysis of the best imported natural mineral waters. We are glad to find that this subject is receiving the attention of a well-known British firm, as without doubt our present knowledge of the therapeutic value of natural mineral waters warrants us in believing that many of them can be so exactly imitated by the chemist that identical results are certain to accrue from their use. It must not, however, be forgotten that the beneficial results which have been obtained at continental spas have been derived not only from the regular and systematic drinking of the waters, but also from the change of scene and environment which often plays the important part in effecting a permanent cure in the condition of the patient that resorts to such treatment. The present system of importing such spa waters in bottles to this country has, however, established the fact that many of these natural waters do act upon the system in a definite and well-ascertained manner apart from any other determining conditions, so that the waters alone are to be recommended in many cases. Messrs. Macfarlan, of Edinburgh, have endeavoured in the Holyrood table water to supply economically an artificial water, which contains those mineral salts which are present in some of these natural mineral waters of the continent, and we have no hesitation in saying that this water can take the place of those imported waters which, in chemical composition, agree with it. As it is possible to regulate the manufacture so that each bottle contains an accurate dose of the several constituent salts, we hope that this attempt of Messrs. Macfarlan will be associated with the commercial success which the idea involves. The samples of the Holyrood water which we have analysed were, on examination, found to be bright, clear, and moderately effervescent. The amount of carbonic acid differed very much in the different bottles, and it would seem that more uniform results could be obtained if, instead of corks, some form of screw cap was employed. The quantity of carbonic acid present was, however, in each case more than sufficient to fully saturate the water at the ordinary temperature. The taste was agreeably saline, giving a distinct alkaline after-taste in the mouth. The total solids found on evaporating a portion of the water amounted to 247·0 parts per 100,000, showing that the water had been impregnated with a considerable quantity of saline constituents; and we should, from this result, rather prefer to describe the water as a mineral water prepared artificially than as a table water, since we could only recommend a water containing this large amount of saline matter, whatever be its nature for general consumption, in those cases in which beneficial results have attended the use of such highly charged natural mineral waters. Our analysis has been confined to a determination of the amount of lime, magnesia, sulphuric acid, and chlorine as chlorides present in the water, and from these determinations the approximate composition of the water as shown below has been derived, based upon the assumption warranted by the temporary hardness of the water that the calcium and magnesium salt present was in the condition of bicarbonate.

ANALYSIS IN PARTS PER 100,000.

| | |
|---|---|
| Sodium sulphate... | 94·57 |
| Calcium carbonate | 29·64 |
| Magnesium carbonate | 28·35 |
| Sodium carbonate | 45·60 |
| Sodium chloride... | 55·19 |
| | 253·35 |

From the total amount of solids calculated in this way it will be seen that the above results approach very closely to the total amount of saline matter actually present in the water, so that the above analysis approximates very closely to the actual constituents of this water. From the results of the analysis it will also be readily seen that the water may be described as a mild alkaline slightly aperient water, and as such should have a wide field of usefulness.

### MALT EXTRACT.

(THE DISTILLERS' COMPANY (LIMITED), EDINBURGH.)

We were favoured by a sample of this company's first trial malt extract, and our analyst has reported on it as follows: The sample submitted to analysis was a brown fluid extract having a very sweet, agreeable taste, with only a slight malty flavour. On analysis it yielded the following figures:—

| | |
|---|---|
| Water ... | 18·7 |
| Maltose | 58·01 |
| Ash ... | 1·66 |

This extract is characterised by the high ash, as most extracts have ashes ranging between 1·1 and 1·4. The maltose is somewhat lower than in the first-class extracts, but there are many on the market with maltose as low as 55 per cent. The diastatic power was satisfactory, as the extract digested its own weight of arrowroot starch in less than ten minutes. The sample was free from any musty flavour or smell, and we can, therefore, recommend it as a rival to other similar preparations already before the public.

